# Implementation of a Phase Synchronization Scheme Based on Pulsed Signal at Carrier Frequency for Bistatic SAR

**DOI:** 10.3390/s20113188

**Published:** 2020-06-04

**Authors:** Yafeng Chen, Da Liang, Haixia Yue, Dacheng Liu, Xiayi Wu, Heng Zhang, Yuanbo Jiao, Kaiyu Liu, Robert Wang

**Affiliations:** 1 Space Microwave Remote Sensing System Department, Aerospace Information Research Institute, Chinese Academy of Sciences, Beijing 100094, China; chenyf@aircas.ac.cn (Y.C.); liangda16@mails.ucas.edu.cn (D.L.); yuehx@aircas.ac.cn (H.Y.); dcliu@mail.ie.ac.cn (D.L.); wuxy@aircas.ac.cn (X.W.); zhangheng@aircas.ac.cn (H.Z.); jiaoyb@aircas.ac.cn (Y.J.); yuwang@mail.ie.ac.cn (R.W.); 2 School of Electronic, Electrical and Communication Engineering, University of Chinese Academy of Sciences, Beijing 100039, China

**Keywords:** phase synchronization, pulsed signal, carrier frequency, bistatic SAR

## Abstract

Phase synchronization is one of the key technical challenges and prerequisites for the bistatic synthetic aperture radar (SAR) system, which can form a single-pass interferometry system to perform topographic mapping. In this paper, an advanced phase synchronization scheme based on a pulsed signal at carrier frequency is proposed for a bistatic SAR system and it is verified by a ground validation system. In the proposed phase synchronization scheme, the pulsed signal at carrier frequency is used for phase synchronization link, and it is exchanged by virtue of a time slot between radar signals. The feasibility of the scheme is proven by theoretical analysis of various factors affecting the performance of phase synchronization, and the reliability of the scheme is verified by the test results of the ground validation system.

## 1. Introduction

**Figure 1:**
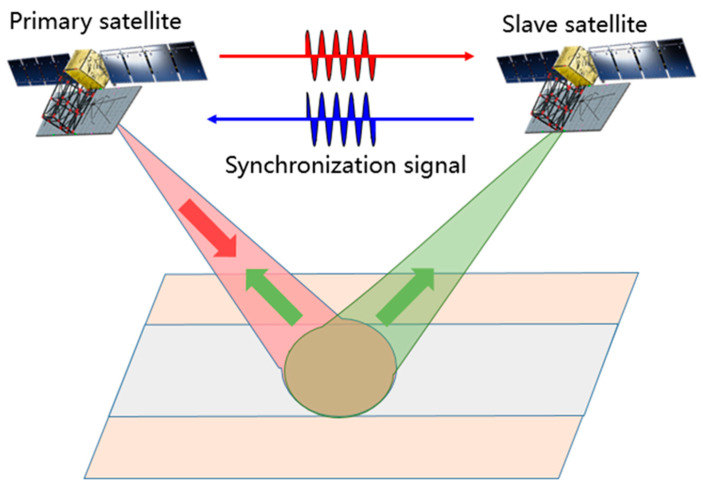
Example of bistatic synthetic aperture radar BiSAR stripmap mode.

Synthetic aperture radar (SAR) is a fast-developing tool for Earth observation in use for the last 40 years [1,2]. Because of its outstanding advantages of all-weather and all-day observation of the Earth, it has been widely used in military reconnaissance, land resources surveys, disaster monitoring, ocean monitoring and yield estimation of crops, surveying and mapping and so on [3,4,5]. In recent years, bistatic SAR (BiSAR) systems have attracted more and more interest as they have various unique features, which are characterized by the different locations of the transmitter and receiver, flexible baseline configurations, costs saving using existing systems as a source of opportunity with several receive-only systems and the possibility to generate highly accurate digital elevation models (DEM) using bistatic interferometry [6,7,8,9]. The BiSAR stripmap mode is shown in Figure 1, which can be used for generating a DEM of the Earth’s surface. In this mode, the primary satellite transmits the radar signal to the ground, and then the echoes are received by the primary satellite and the slave satellite.

However, the unique characteristics of BiSAR also add new challenges in technology implementation, such as measurement, control of the satellite platforms, the synchronization of the time, beam and phase, and BiSAR imaging formation algorithms and so on. Among these challenges, phase synchronization is one of the most critical factors for BiSAR interferometry [10,11,12]. Phase synchronization can be realized by exchanging the synchronization signal between two platforms in BiSAR systems, as has been investigated in [13,14,15,16,17,18,19,20,21,22]. The pulse alternate synchronization scheme is proposed in and has been used for the TanDEM-X [16,18]. Each satellite in the TanDEM-X mission is equipped with six synchronization antennas used for transmitting and receiving the synchronization signal [18,19,20]. The phase synchronization accuracy in the TanDEM-X mission is less than 1° [20]. A linear frequency modulation (LFM) signal is used for exchanging. The LFM signal with the same carrier frequency as the transmitted radar signal is used for synchronization in order to simplify the system design. To date, the carrier frequency signal used for synchronization in BiSAR systems has not been demonstrated in the literature. In this paper, we investigate a pulsed signal at carrier frequency used for phase synchronization. From the test results, we can see that the synchronization scheme with the pulsed signal at carrier frequency has good performance. The test results of the synchronization scheme in the paper have guiding significance for the synchronization design of bistatic and multistatic SAR systems.

This paper is organized as follows. In Section 2, the proposed pulsed signal phase synchronization scheme is introduced, including a pulsed synchronization signal exchange strategy and performance analysis. The performance verification of the phase synchronization is analyzed in Section 3. In Section 4, the validation experiment is conducted using the LuTan-1 ground test system. The performance of the proposed synchronization scheme is evaluated in Section 4. Finally, Section 5 concludes this paper with a short summary.

## 2. Pulsed Signal at Carrier Frequency Synchronization Implementation Scheme

In this section, the phase synchronization implementation scheme based on a pulsed signal at carrier frequency is established, which is later used as a basis for deriving quantitative estimates for the performance of the synchronization link.

### 2.1. Synchronization Scheme Description

**Figure 2:**
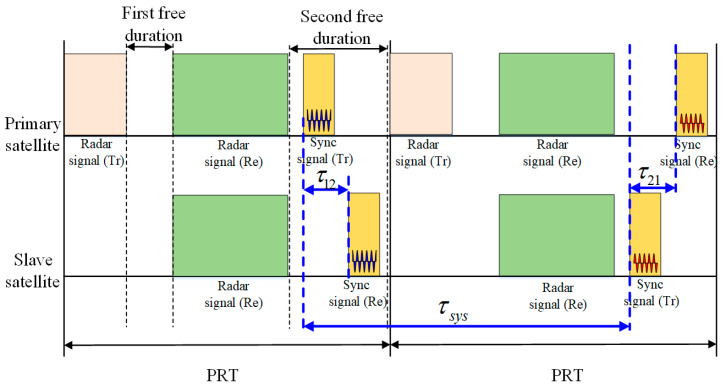
Timing diagrams of synchronization pulse exchange.

In the BiSAR system, frequency deviation and phase noise of the radar carrier are the main factors that cause a phase synchronization error between the primary satellite and the slave satellite. In principle, phase synchronization can be achieved as long as the frequency difference and phase noise signal of the primary satellite and the slave satellite are obtained through the bidirectional transmission of the radar carrier, and the echo received by the slave satellite is compensated by it [22]. In this paper, on the basis of the phase synchronization scheme proposed by Younis et al. [15,16] and applied to the TanDEM-X system with a bidirectional pair of phase synchronization pulses, an improved phase synchronization scheme was proposed for BiSAR satellites, which was based on a pulsed signal at carrier frequency as an uninterruptable bidirectional pair of synchronization pulses. The key points of the scheme are as follows:

- The phase synchronization signal is exchanged by virtue of a time slot between radar signals; thus, the normal work of the BiSAR satellites can be prevented from being interrupted. Time diagrams of the synchronization pulse exchange are shown in Figure 2.
- The primary satellite and the slave satellite demodulate and collect the phase synchronization pulses received, respectively. In the data processing, the compensation phase is extracted by fast Fourier transform (FFT) operation of the pulsed signal, and the echo of the slave satellite is compensated by the compensation phase to complete the phase synchronization.

In the first pulse repetition time (PRT), the primary satellite transmits the synchronization signal to the slave satellite and the slave satellite receives the synchronization signal. Correspondingly, at the next PRT, the slave satellite transmits the synchronization signal to the primary satellite and the primary satellite receives the synchronization signal. The synchronization signal is at carrier frequency; therefore, the phase with a high signal-noise ratio (SNR) can be obtained at the frequency domain by fast Fourier transform (FFT) operation. The processing of the synchronization signal can be summarized as follows:

- Pulse compression by FFT operation. The data are transformed into frequency domain;
- The peak phases are extracted at peak position of amplitude in the compression synchronization data.

### 2.2. Synchronization Scheme Performance Analysis

The frequency of oscillator at the start of data take $t_{0}$ is $f_{i}=f_{0}+\Delta f_{i}$, where i∈{1,2}, which represents the primary satellite and slave satellite. $f_{0}$ represents the nominal frequency, $f_{i}$ is the carrier frequency and $\Delta f_{i}$ denotes frequency offset [23,24,25,26]. The phases of two satellites at time are:

(1) $$\varphi_{i}(t)=2\pi \int_{0}^{t}\left(f_{0}+\Delta f_{i})dt+\varphi_{0i}+n_{\varphi_{i}}(t\right)$$

where $\varphi_{0i}$ is the initial time-independent phase and $n_{\varphi_{i}}(t)$ is the oscillator phase noise.

The demodulated phase $\varphi_{ij}(t)$ available at satellite i for a signal transmitted by satellite j. Thus, the demodulated phase $\varphi_{21}$ is available at the slave satellite for a signal transmitted by the primary satellite [15,16].

(2) $$\begin{array}{ll} \varphi_{21}(t) & =\varphi_{1}(t)-\varphi_{2}(t+\tau_{12})\\ & =2\pi \left(\Delta f_{1}-\Delta f_{2}\right)t-2\pi \left(f_{0}+\Delta f_{1}\right)\tau_{12}\\ & \;+\varphi_{01}-\varphi_{02}+n_{\varphi 1}(t)-n_{\varphi 1}(t+\tau_{12}) \end{array}$$

The output phase when the slave satellite transmits a synchronization pulse at $t=t+\tau_{sys}$ while the primary satellite receives at $t+\tau_{sys}+\tau_{21}$, and the demodulated phase $\varphi_{12}$ is [15,16]:

(3) $$\begin{array}{ll} \varphi_{12}(t)= & 2\pi \left(\Delta f_{2}-\Delta f_{1}\right)\left(t+\tau_{sys}\right)-2\pi \left(f_{0}+\Delta f_{1}\right)\tau_{21}\\ & +\varphi_{02}-\varphi_{01}+n_{\varphi 2}(t+\tau_{sys})-n_{\varphi 1}(t+\tau_{sys}+\tau_{21}) \end{array}$$

**Figure 3:**
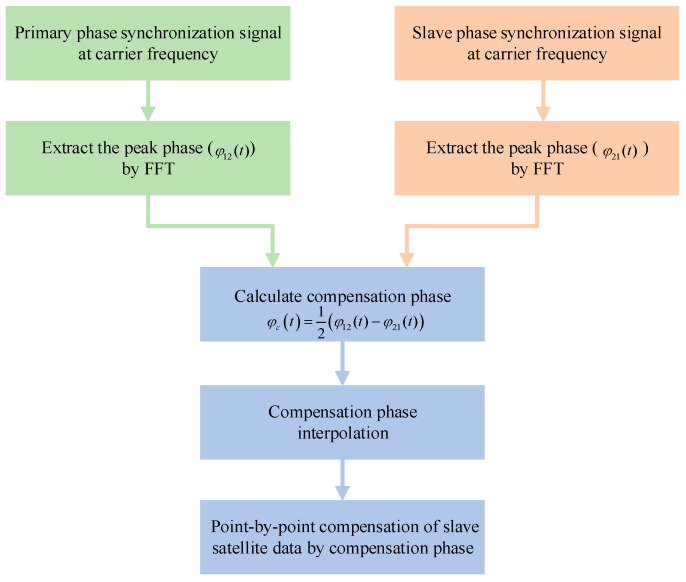
The flow diagram of synchronization signal processing.

The flow diagram for synchronization signal processing is shown in Figure 3 and the compensation phase can be represented as [15,16]:

(4) $$\begin{array}{ll} \varphi_{c}\left(t\right) & =\frac{1}{2}\left(\varphi_{12}(t)-\varphi_{21}(t)\right)\\ & =2\pi \left(\Delta f_{2}-\Delta f_{1}\right)t+\pi \left(\Delta f_{2}-\Delta f_{1}\right)\left(\tau_{12}+\tau_{sys}\right)-\pi f_{D}\tau_{sys}+\\ & \frac{1}{2}\left(n_{\varphi 2}(t+\tau_{sys})+n_{\varphi 2}(t+\tau )-n_{\varphi 1}(t)-n_{\varphi 1}(t+\tau +\tau_{sys})\right)\\ & \approx 2\pi \Delta f(t)t+\pi \Delta f(t)(\tau +\tau_{sys})-\pi f_{D}\tau_{sys}+\varphi_{res} \end{array}$$

where $\Delta f(t)=\Delta f_{2}(t)-\Delta f_{1}(t)$ the frequency is offset between two oscillators, $f_{D}$ is the Doppler frequency due to the relative velocity between the two satellites and $\varphi_{res}$ is residual phase, which includes the initial phase difference between two oscillators, oscillator noise phase error and hardware system phase error.

The time synchronization error can be up to 10^−8^ s, so in a short time, it can be considered stable [27,28,29]. As shown in Figure 3, phase unwrapping starts the primary and secondary synchronization phases, and after phase unwrapping, interpolation is performed to obtain a synchronization phase consistent with the phase dimension of the echo and the compensation phase can be used to compensate the satellite data point-by-point.

### 2.3. Comparison between Pulsed Signal at Carrier Frequency and LFM Signal

Here, a comparison between a pulsed signal at carrier frequency and an LFM signal is given. The LFM signal $s_{LFM}(\tau )$ is written as follows:

(5) $$s_{LFM}(\tau_{k})=rect(\frac{\tau_{k}}{T})A\exp(j\pi K_{r}\tau_{k}{}^{2})$$

where rect() is the rectangular function, A is the amplitude, $K_{r}$ is the frequency modulation rate and T is the pulse width of the LFM signal. $\tau_{k}$ is fast time, where k = 1,2,···, $\left[TF_{s}\right]$ and $F_{s}$ is the sampling frequency. Suppose the bandwidth of the LFM signal $s_{LFM}(\tau )$ is B. In the receiving signal, suppose the average noise power is $n_{niose}$ and there are $N_{r}$ samples. Therefore, the total noise power $P_{niose}$ of $N_{r}$ samples can be expressed as

(6) $$P_{noise}=N_{r}n_{noise}$$

After matching filtering in the frequency domain, the LFM signal $s_{LFM}(\tau )$ is compressed. The peak power in the peak position can be written as

(7) $$P_{LFM}=A^{2}TB$$

In addition, the total noise power after filtering can be expressed as $P_{noise,filter}=N_{r}n_{noise}B/F_{s}$. The average noise power after filtering is $n_{noise,filter}=n_{noise}B/F_{s}$. Therefore, the SNR of the LFM signal after matching filtering is

(8) $$SNR_{LFM}=\frac{P_{LFM}}{n_{noise,filter}}=\frac{A^{2}BT}{n_{noise}B/F_{s}}=\frac{A^{2}TF_{s}}{n_{noise}}$$

Suppose the pulse signal at carrier frequency (after demodulation) is written as follows:

(9) $$s_{carrier}(\tau_{k})=\mathrm{rect}(\frac{\tau_{k}}{T})A\exp(-j2\pi \Delta f(t)\tau_{k})$$

For the purpose of comparison, the signal $s_{carrier}(\tau )$ has the same amplitude *A* and pulse width T as the LFM signal. After FFT operation, the peak power in the peak position can be expressed as follows:

(10) $$P_{carrier}=N_{r}A^{2}TF_{s}$$

The average noise power in the frequency domain can be written as $n_{noise,f}=N_{r}n_{noise}$. Therefore, the SNR of the $s_{carrier}(\tau )$ is given by

(11) $$SNR_{carrier}=\frac{P_{carrier}}{n_{noise,f}}=\frac{N_{r}A^{2}TF_{s}}{N_{r}n_{noise}}=\frac{A^{2}TF}{n_{noise}}$$

It can be seen from Equations (8) and (11) that the SNRs of the LFM signal and pulse signal at carrier frequency are the same.

## 3. Pulsed Signal at Carrier Frequency Phase Synchronization Scheme Verification

In order to evaluate the performance of the pulsed signal at carrier frequency synchronization scheme, the test experiment based on the ground validation system of the LuTan-1 is performed. As mentioned above, the LFM signal is used for synchronization in the LuTan-1 mission. However, with some modification, the ground validation system of LuTan-1 can be used for demonstrating the pulsed signal at carrier frequency synchronization scheme. In this section, the hardware system is introduced. The test experiment is described in detail.

### 3.1. Synchronization Scheme Performance Analysis

**Figure 4:**
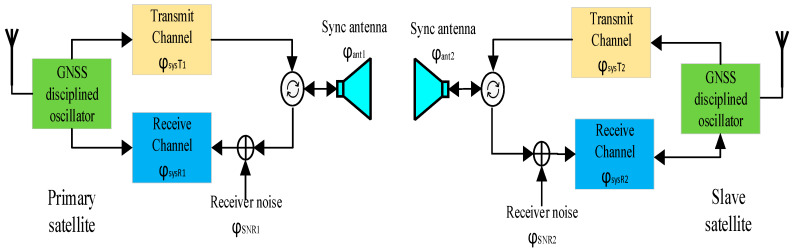
The hardware structure of the phase synchronization link.

The hardware structure diagram of the phase synchronization link is illustrated in Figure 4. The system consists of a global navigation satellite system (GNSS) disciplined oscillator, a transmit channel, a receive channel and a synchronization antenna. The GNSS disciplined oscillator employs disciplined rubidium clocks to provide a time frequency signal for the reference frequency source module. The GNSS disciplined oscillator combines the excellent short-term stability characteristics of quartz crystal oscillators with the long-term stability characteristics of GPS signals [25,30]. In the previous test of the ground validation system of LuTan-1, an oscillator with 10^−7^ accuracy was used and the experiments presented in the paper were conducted. However, when satellites are in orbit, a GNSS disciplined oscillator is used in the system and accuracy can reach 10^−11^.

The purpose of extracting the compensated phase is to obtain the phase synchronization error caused by the frequency deviation of the primary and slave satellites and other phase noise. The other phases introduced in this process will become the interference phase that affects the phase synchronization performance. It can be seen from Figure 4 that during the phase synchronization pulse transmission, the factors that cause the phase change include: primary satellite and slave satellite frequency offset Δf(t), the phase jitter of transmit and receive channels $\varphi_{sysT_{i}}$ and $\varphi_{sysR_{i}}$ and the phase jitter caused by receiver noise is $\varphi_{SNR_{i}}$. The phase jitter caused by synchronization antenna is $\varphi_{ant_{i}}$ and the phase jitter caused by the Doppler effect of relative motion of the primary satellite and slave satellite is 2π△d/λ, where i=1 represents the primary satellite and i=2 represents the slave satellite.

The compensation phase interpolation can be represented as [25]:

(12) $$\begin{array}{ll} \varphi_{c}\left(t\right) & =\frac{1}{2}\left(\varphi_{12}(t)-\varphi_{21}(t)\right)\\ & =2\pi \left(\Delta f_{2}-\Delta f_{1}\right)t+\pi \left(\Delta f_{2}-\Delta f_{1}\right)\left(\tau_{12}+\tau_{sys}\right)-\pi f_{D}\tau_{sys}\\ & +\;\frac{1}{2}\left[n_{\varphi 2}(t+\tau_{sys})+n_{\varphi 2}(t+\tau )\right]-\frac{1}{2}\left[n_{\varphi 1}(t)+n_{\varphi 1}(t+\tau +\tau_{sys})\right]\\ & +\frac{1}{2}\Delta \varphi_{sys}(t)+\frac{1}{2}\Delta \varphi_{ant}(t)\;+\frac{1}{2}\Delta \varphi_{SNR}(t) \end{array}$$

where $\Delta \varphi_{sys}(t)$ represents the difference in transmit/receive channel phase, $\Delta \varphi_{ant}(t)$ the influence of the phase of the antenna pattern and $\Delta \varphi_{SNR}\left(t\right)$ denotes the difference of phase error caused by receiver noise.

According to Equation (12) of the analysis, the phase error introduced by the synchronous antenna pattern is much smaller than 0.01° and can be ignored. The phase jitter of the BiSAR transmit and receive channels can generally be controlled within 0.01°, and the impact on the phase synchronization performance is also small, having little effect on the phase synchronization performance. The receiver noise phase jitter, which can be regarded as the total synchronization link error caused by the receiver noise and sampling, after azimuth compression, can be further reduced. From Equation (12), it can be seen that the first term is the phase difference formed by the frequency deviation of the primary satellite and the slave satellite, where the accuracy of the atomic clock frequency used on GNSS is more than 10^−12^ orders of magnitude [25]. It can be compensated by the frequency and time difference data in the primary and slave satellites. The fifth and sixth terms are the phase difference formed by the phase noise, which can be obtained from the phase synchronization signal as an FFT algorithm.

### 3.2. Hardware Implementation of Phase Synchronization Scheme

**Figure 5:**
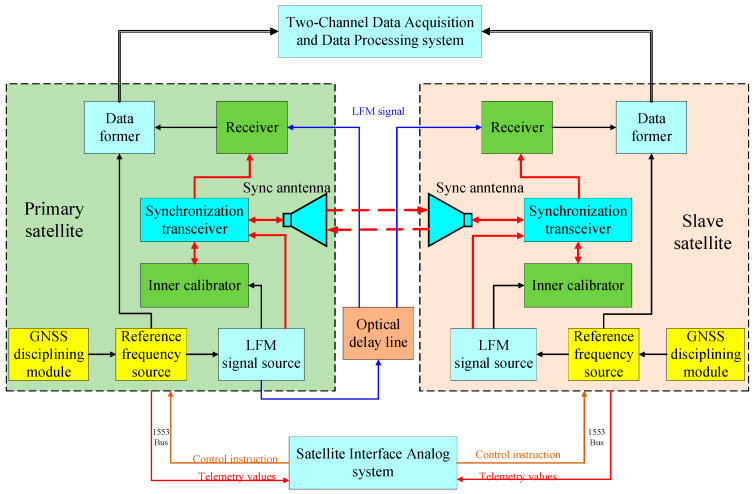
The structure block diagram of the ground test system.

In accordance with the above phase synchronization scheme, the L-band radar central electronic equipment with the phase synchronization function of the primary satellite and the slave satellite is developed, and the ground verification test of phase synchronization is carried out. The structure block diagram of the LuTan-1 ground test system is in Figure 5.

In the test experiment, two satellites are placed in a microwave anechoic chamber. An optical delay line is used to simulate the echo-receiving process for the two satellites. The primary satellite transmits the radar signal. The radar signal passes through the optical delay line and then is received by the primary satellite and slave satellite. After range compression, the peak phases of the radar echo signals, which can be referred to as the reference phases, are extracted in the peak position for primary satellite and slave satellite, respectively. The synchronization signals are exchanges between two synchronization antennas. The phase synchronization scheme performance of the system can be obtained by analyzing the experimental data with a 120 s data take.

As shown in Figure 5, the GNSS disciplined oscillator module employs a disciplined rubidium clock to provide a time frequency signal for the reference frequency source. The LFM signal source is composed of three parts: a programmable digital chirp baseband generation module, quadrature modulation and power amplification and a multimode output module. According to the programming control signal, a baseband linear frequency modulation signal with a certain bandwidth, a certain pulse width and a specified slope that meets the needs of the system is generated. Mode combination gating filtering and power amplification to obtain RF chirp signals are used to meet the requirements in time domain, frequency domain, modulation domain and signal power. The former data module completes the analog-to-digital conversion (ADC) of the intermediate frequency. An optical delay line is used to simulate the echo-receiving process for the two satellites. The primary satellite transmits the radar signal. The radar signal passes through the optical delay line and then is received by the primary satellite and slave satellite.

The test system consists of the satellite interface analog system, the primary satellite central electronic equipment, the slave satellite central electronic equipment, the two-channel data acquisition and data processing system and the quadrifilar helix antenna used for the LuTan-1 phase synchronization link. The experimental parameters are shown in Table 1.

The satellite interface ground analog system sends control instructions to the central electronic equipment of the two satellites through a 1553 data bus and obtains their important telemetry values in real time to ensure the normal operation of the two satellites. The two-channel data acquisition and data processing system collects the echo signal and synchronization signal at the same time, calculates the compensation phase and compensates the echo data of the slave satellite point-to-point. The central electronic equipment of the SAR system consists of a signal generation unit, a transmit/receive unit and an inner calibration unit. The LFM signals transmitted by the primary satellite pass through the optical delay line, and then they are received by the two satellites to simulate the echo-receiving process. The synchronization pulses are transmitted using the quadrifilar helix antenna between the two satellites.

**Figure 6:**
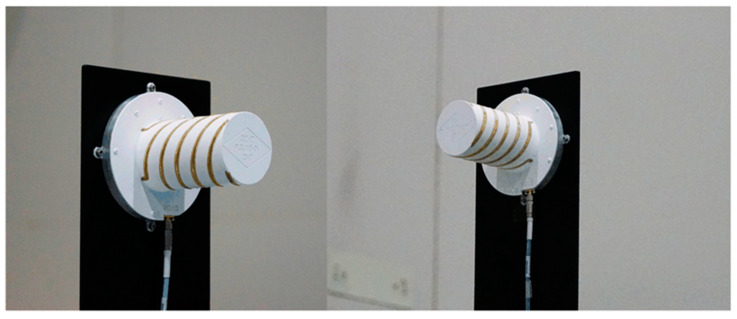
The quadrifilar helix antenna used for synchronization link.

The quadrifilar helix antenna used for synchronization link in the test system is shown in Figure 6. This quadrifilar helix antenna incorporated broadband antennas, a wide beam, circular polarization, light weight and low-profile design. The actual measurement results show that the antenna standing wave bandwidth can reach 61.1%, and the 3 dB beam width is greater than 120°.

## 4. Discussion

### 4.1. Theoretical Error Analysis

In the test system, the GNSS disciplined oscillator module employs a disciplined rubidium clock to provide a time frequency signal. The ground GNSS disciplined oscillator has added the function of correcting phase every 10 s. For LuTan-1 synchronization, where the maximum value can be set to 1/2 PRF, the system works at a high phase synchronization frequency, so as to avoid a phase synchronization precision error and ensure phase synchronization accuracy. The receiver noise, which consists of thermal noise and the noise collected by the synchronization, will introduce both amplitude and phase fluctuations to the synchronization signal and reduce the accuracy of phase synchronization [15].

The total phase variance in phase synchronization can be written as [15]

(13) $$\sigma_{link}^{2}=\sigma_{i}^{2}+\sigma_{a}^{2}+\sigma_{f}^{2}+\frac{1}{2}\sigma_{SNR}^{2}$$

where $\sigma_{i}^{2}$ denotes the interpolation variance, $\sigma_{a}^{2}$ denotes the aliasing variance, $\sigma_{f}^{2}$ is filter mismatch variance and $\frac{1}{2}\sigma_{SNR}^{2}$ is the receiver noise variance. In the proposed synchronization scheme, the synchronization rate can be very high. As a result, the interpolation variance and aliasing variance are very small and can be ignored in the analysis. In addition, supposing there is no filter mismatch error, $\sigma_{f}^{2}$ is also ignored for the sake of simplification. Therefore, the total error variance can be expressed as follows [15]

(14) $$\sigma_{link}^{2}=\frac{1}{2}\sigma_{SNR}^{2}=\frac{1}{4f_{syn}\mathrm{SNR}}\int_{-f_{syn}/2}^{f_{syn}/2}{\left|H_{syn}(f)\right|}^{2}df$$

where $f_{syn}$ is the synchronization rate. $H_{syn}(f)$ is the transfer function and can be written as:

(15) $$H_{syn}(f)=\frac{1}{\exp(-j\pi f\tau_{sys})\cos(\pi f\tau_{sys})}$$

**Figure 7:**
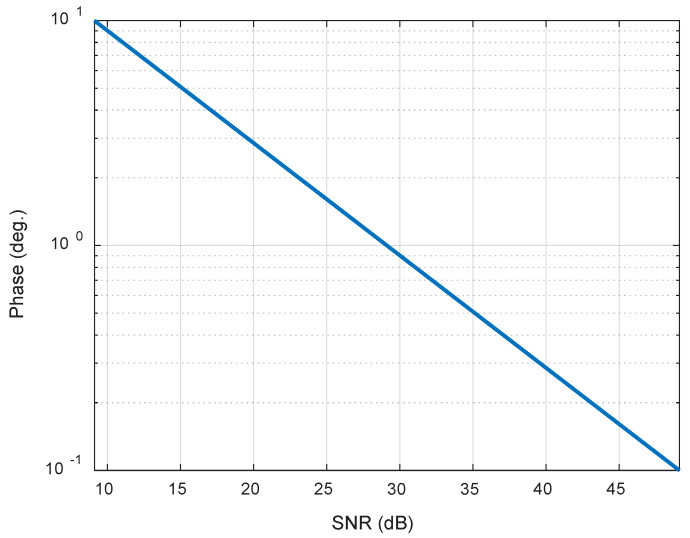
The standard deviation (STD) of phase errors versus signal-noise ratio (SNR).

Using the error model mentioned above, the theoretical bounds are given in Figure 7.

Therefore, as shown in Figure 7, if we want the SNR of the phase synchronization signal to be around 30 dB, the phase accuracy is within 1°.

### 4.2. Results of Test Experiment

**Figure 8:**
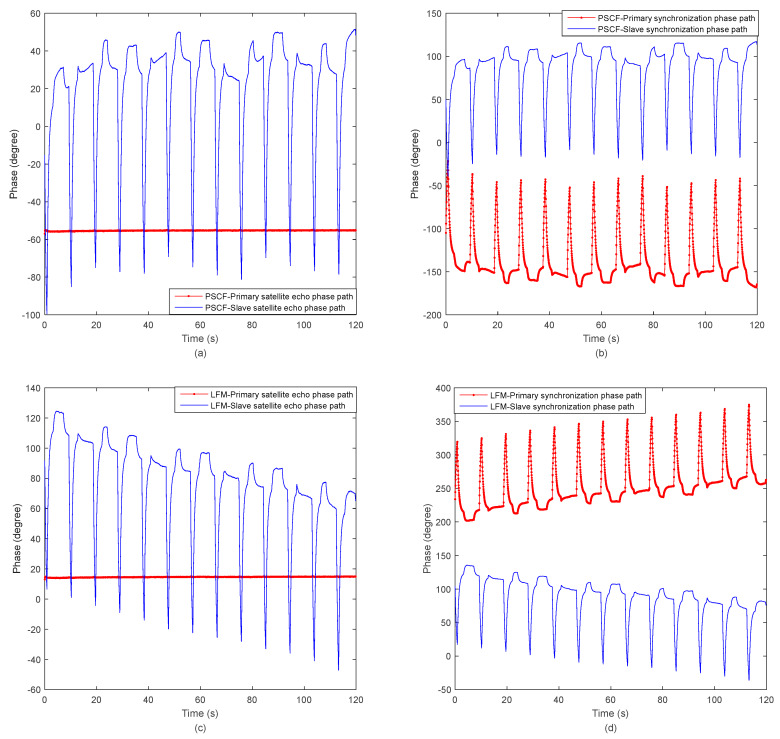
The echo phase and synchronization phase of the two satellites. (**a**) pulsed signal at carrier frequency (PSCF)-the radar signal phases of the two satellites; (**b**) PSCF-the synchronization phases of the two satellites; (**c**) linear frequency modulation (LFM)-the radar signal phases of the two satellites; (**d**) LFM-the synchronization phases of the two satellites.

Figure 8a shows that the primary satellite works spontaneously, its phase basically does not change with time, while the signal received by the slave satellite is sent by the primary satellite, and its phase changes with azimuth time. In the ground test system experiment, the system works in the BiSAR mode. Further, the primary satellite and slave satellite respectively use two sets of GNSS disciplined oscillators and reference frequency sources. The GNSS disciplined oscillator employs a disciplined rubidium clock to provide a time frequency signal for the reference frequency source module, and the multiple working frequency signals are generated by the reference frequency source. The primary satellite is self-transmitting and self-receiving, and since there is no synchronization error, the phase change of the recorded radar signals is mainly introduced by the system instrument, which can be ignored. The slave satellite is only-receiving, and the radar echo signals’ phase change is introduced by the GNSS disciplined oscillator module. Because the ground GNSS disciplined oscillator has added the function of correcting phase every 10 s, the radar echo phase of the slave satellite is periodically calibrated, as shown in the blue curve in Figure 8a. The primary satellite synchronization phase path and slave satellite synchronization phase path is shown in Figure 8b. The LFM-phase synchronization scheme, the radar signal phases and the synchronization phases of the two satellites are shown in Figure 8c,d, respectively.

**Figure 9:**
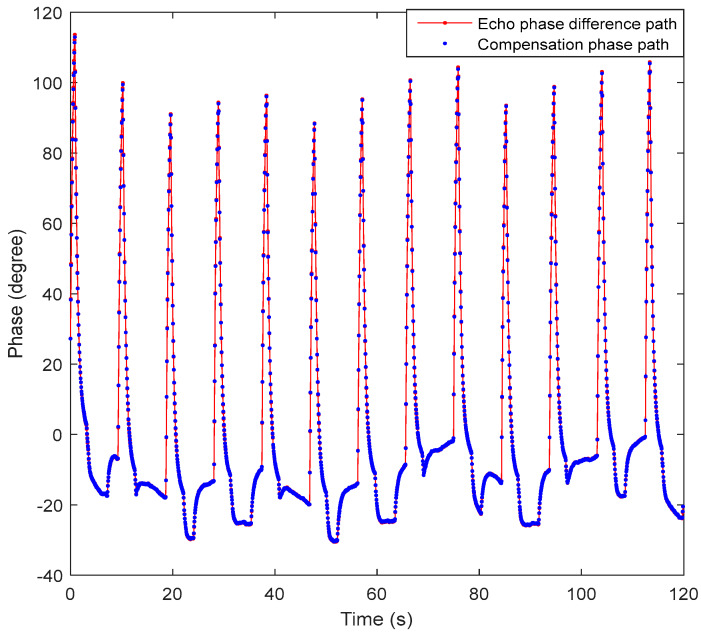
The echo phase difference and compensation phase of the two satellites.

Figure 9 shows the echo phase compensation phase of the two satellites and the compensation phase path of the pulsed signal at carrier frequency (PSCF)-phase synchronization scheme. The compensation phase path is very close to the echo phase difference path. The residual phase is obtained after the compensation, which can be seen in Figure 9.

**Figure 10:**
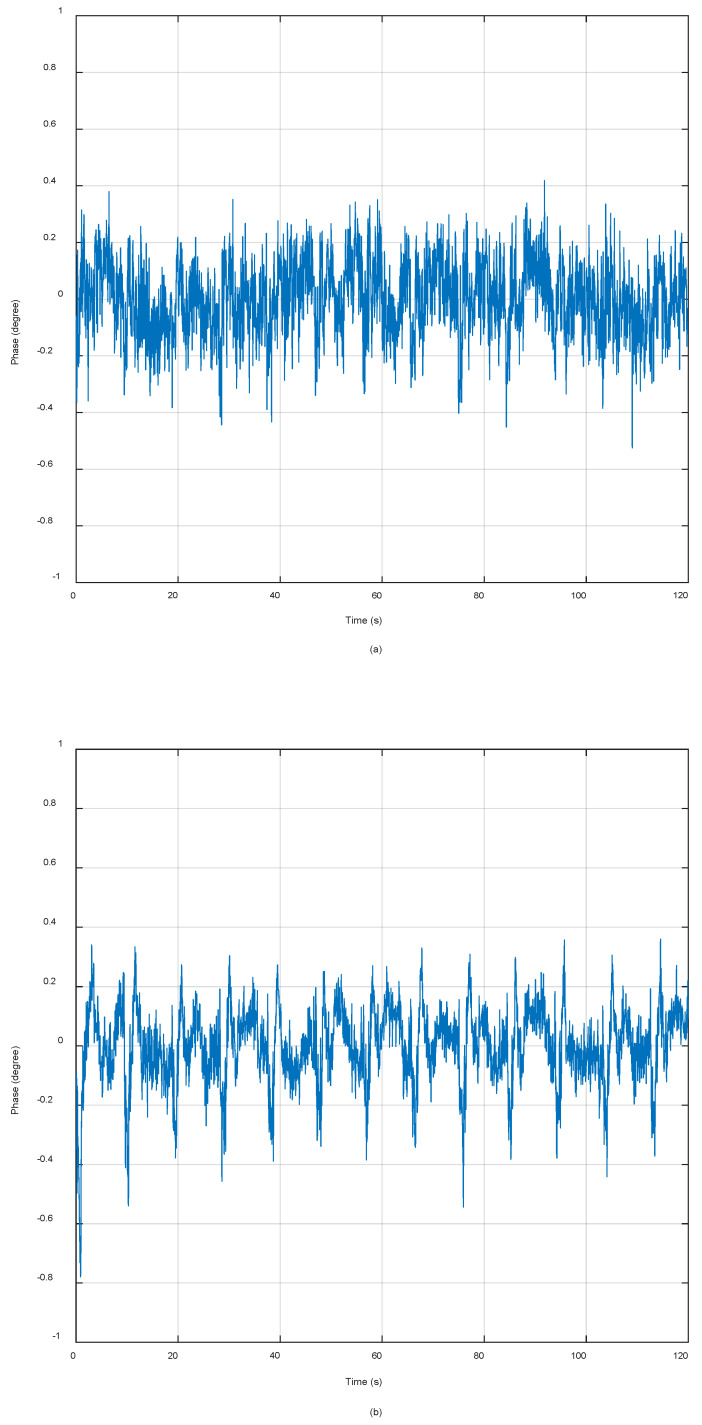
The residual phase error after phase synchronization compensation. (**a**) Residual phase of LFM-phase signal scheme; (**b**) residual phase of pulsed signal at carrier frequency (PSCF)-phase signal scheme.

Figure 10 shows the echo phase difference and compensation phase of the two satellites. The LFM-phase signal scheme residual phase is obtained after the compensation, which can be seen in Figure 10a, the standard deviation (STD) of the residual phase value is 0.978° and the peak–peak value is 0.524°. The pulsed signal at carrier frequency (PSCF) phase signal scheme residual phase is obtained after the compensation, which can be seen in Figure 10b, the standard deviation (STD) of the residual phase value is 0.780° and the peak–peak value is 1.139°. The comparison results of LFM-phase signal and PSCF-phase signal phase synchronization schemes are shown in Table 2. It can be seen that the PSCF-phase signal is slightly worse than the linear frequency modulation during the short-term startup of the BiSAR, but it can meet the requirements of accuracy. Moreover, the non-interrupted PSCF at carrier frequency synchronization strategy has simple requirements for system design and high system reliability, which have guiding significance for the synchronization design of bistatic and multistatic SAR systems.

## 5. Conclusions

In this paper, an improved scheme of non-interrupted synchronization pair alternates based on pulsed signal at carrier frequency phase synchronization signal is implemented and verified in the LuTan-1 ground test system. The feasibility of using a pulse signal at a carrier frequency as a phase synchronization signal and using an FFT algorithm to rapidly extract the synchronization error of two satellites and the phase synchronization in imaging are analyzed. In addition, the performance of the proposed scheme is verified by test results of the ground validation system. In order to further improve the BiSAR system reliability and reduce system complexity, compared with the LFM-phase synchronization scheme, the PSCF-phase synchronization scheme has an independent synchronization path to correct phase changes during the synchronization operation. Finally, the PSCF-phase synchronization system can fulfil the synchronization accuracy requirement for the BiSAR system.

## Figures and Tables

**Table 1:** Experimental parameters.

Parameter	Value
Carrier frequency	1.26 GHz
Signal bandwidth	80 MHz
Sampling rate	360 Mbps
SNR	30 dB
PRF	3000 Hz
Total recorded pulse number	360,000

**Table 2:** The comparison results of two phase synchronization schemes.

Device	Peak-Peak	Standard (1σ)
LFM-phase signal	0.978°	0.524°
PSCF-phase signal	1.139°	0.780°

## Abbreviations

The following abbreviations are used in this manuscript:

SAR	Synthetic aperture radar
BiSAR	Bistatic synthetic aperture radar
LFM	Linear frequency modulation
PRT	Pulse repetition time
PRF	Pulse repetition frequency
GNSS	Global navigation satellite system
FFT	Fast Fourier transform
DEM	Digital elevation models
PSCF	Pulsed signal at carrier frequency
STD	Standard deviation
SNR	Signal-noise ratio
